# Undiagnosed Term Abdominal Pregnancy in a District-Level Hospital of a Developing Country: A Miracle Baby

**DOI:** 10.7759/cureus.35092

**Published:** 2023-02-17

**Authors:** Win Win Than, DG Marshitah Binti PG Baharuddin, M Tanveer Hossain Parash, Aung Mra

**Affiliations:** 1 Obstetrics and Gynaecology Unit, Faculty of Medicine, AIMST University (Asian Institute of Medicine, Science and Technology), Bedong, MYS; 2 Department of Obstetrics and Gynaecology, Faculty of Medicine and Health Sciences, Universiti Malaysia Sabah, Kota Kinabalu, MYS; 3 Department of Biomedical Sciences, Faculty of Medicine and Health Sciences, Universiti Malaysia Sabah, Kota Kinabalu, MYS; 4 General Surgery, Retired Consultant, Yangon, MMR

**Keywords:** haemorrage, district hospital, developing country, placental attachment, abdominal pregnancy

## Abstract

Term abdominal pregnancy is a sporadic ectopic pregnancy associated with high maternal and perinatal morbidity and mortality. As symptoms are non-specific and resemble those of other ectopic pregnancies, early diagnosis is the major challenge in poor health setups. A 24-year-old primigravida at 38 weeks gestation was planned to undergo a cesarean section for the transverse lie. Abdominal pregnancy was accidentally discovered during the cesarean section, and a healthy, normal baby boy was delivered. The placenta was attached to the greater omentum, so its removal required omentectomy without compromising the blood supply to the bowels. Both patient and her baby boy were discharged on the seventh day without complications. No congenital anomalies were detected in the baby. In a term abdominal pregnancy, the most significant challenges are the control of bleeding and the decision on placenta removal, followed by prompt delivery of the fetus. Therefore, along with the gynecologist, the availability of trained personnel, such as anesthetists, pediatricians, and general surgeons, is necessary for a successful management outcome.

## Introduction

Term abdominal pregnancy is a sporadic type of ectopic pregnancy (ep). The incidence of abdominal pregnancy is 1:10,000 to 1:30,000 pregnancies [1]. Despite advances in ultrasound imaging in recent years and widespread application in modern obstetrics, the incidence of abdominal pregnancies remains at 1% of all ep. Because of the lack of proper guidelines for managing term abdominal pregnancy, it is associated with a 0.5% to 18% maternal mortality rate and a perinatal mortality rate of 40% to 95% if it is not diagnosed at an early gestational age [1].

Abdominal pregnancy is the embryo's implantation in the peritoneal cavity. Abdominal pregnancy can be either primary or secondary. In the primary variation, implantation occurs directly into the peritoneal cavity. In the secondary variation, implantation occurs in the fallopian tube at first, then aborts through the fimbrial end of the fallopian tube, and subsequently implants itself in the peritoneal cavity. The pregnancy is defined as advanced abdominal pregnancy that has continued beyond 20 weeks. Up to 48% of abdominal pregnancy cases could be diagnosed before delivery, making it possible to prepare for and anticipate hemorrhage and referral to the neonatal team to optimize the outcome of the abdominal pregnancy [2]. In an abdominal pregnancy, perinatal mortality was reported as 72% [3].

We report a case of advanced-term abdominal pregnancy that presented to a district hospital in a poor resource country with a favorable outcome for both mother and baby.

## Case presentation

A 24-year-old primigravida was under the care of a senior medical officer throughout her pregnancy at a private clinic. At 38 weeks gestation, she was apparently stable and her vital signs were normal. No abnormality was detected on general and systemic examination. The abdominal examination revealed a transverse lie, a symphysis-fundal height of 29 cm, a fetal heart rate of 136 beats per minute, and no uterine contractions. A vaginal examination revealed an empty pelvis and no cervical dilation. She was later admitted to a district hospital with the plan of the cesarean section indicated by the transverse lie. The district hospital had no blood bank or ultrasound machine for imaging investigations.

**Figure 1:**
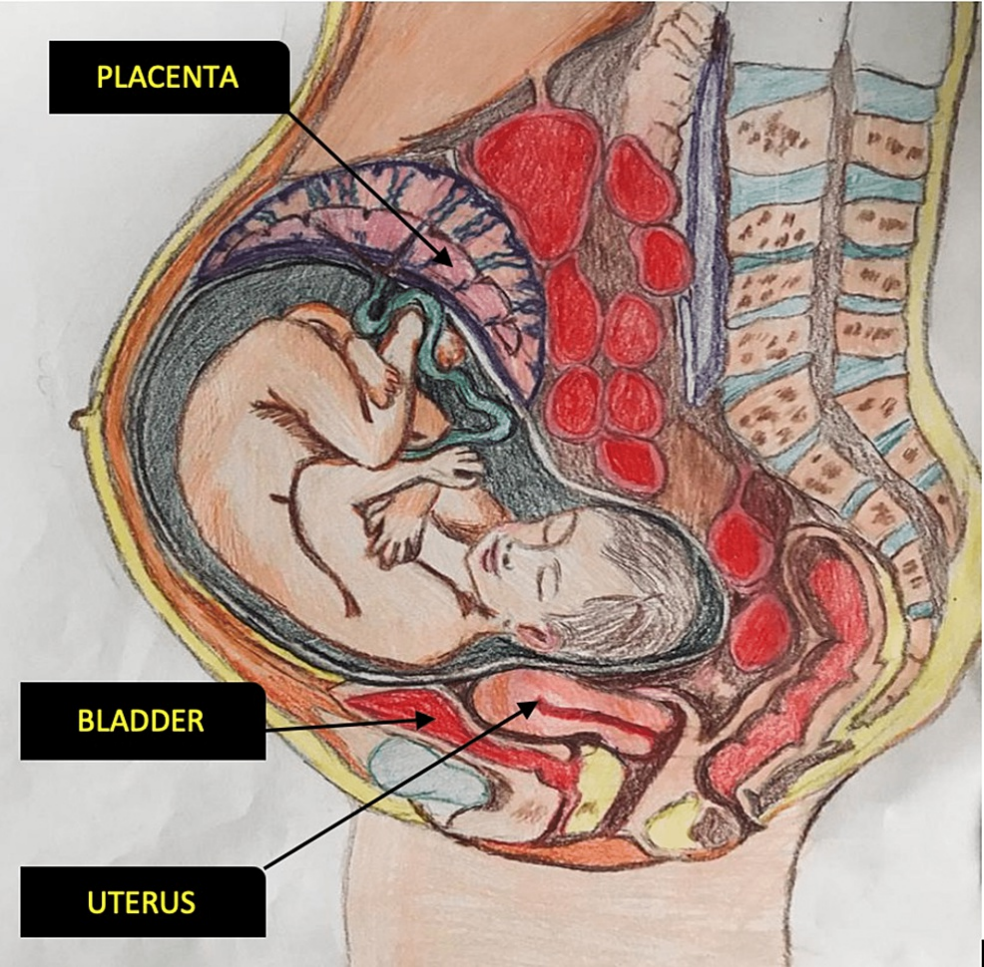
Schematic representation of the term abdominal pregnancy

After obtaining informed consent, the cesarean section was commenced by the right paramedian approach under spinal anesthesia. Once the peritoneum was opened, there was a massive sac-like structure in the peritoneal cavity and a regular-sized uterus. Further exploration revealed an amniotic sac containing a fetus inside the peritoneal cavity. The uterus was bulky, and both ovaries and fallopian tubes were normal (Figure 1). Once the amniotic sac was ruptured, a baby boy weighing 6 pounds and 12 ounces was delivered. The newborn had a vigorous cry with APGAR (Appearance, Pulse, Grimace, Activity, and Respiration) scores of 8 (at 1 minute) and 10 (at 5 minutes) and was referred to a pediatrician for further management.

**Figure 2:**
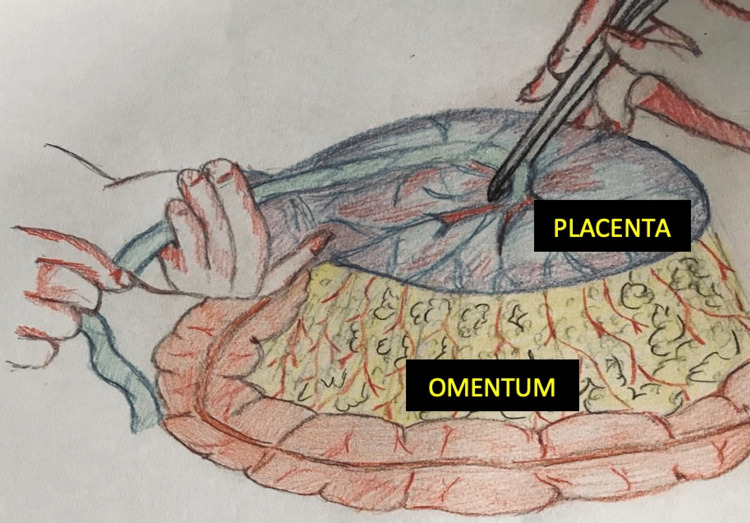
Schematic representation of term placental attachment to the greater omentum

After the baby was delivered safely, the placental attachment was further explored. The placenta appeared to be attached to the greater omentum. When the placenta was lifted for a better view, its attachment to the omentum was traceable and confirmed (Figure 2). The whole placenta removal involved an omentectomy since it got its blood supply from the omentum. Bleeding points were secured, and the abdominal closure was done in layers. The blood loss was estimated to be 200 ml.

Upon retrospective history, it was found that the patient suffered from frequent abdominal pains throughout her pregnancy, and fetal lie and presentation kept changing at each antenatal visit. The patient and her baby were discharged uneventfully on the seventh day, without complications. No congenital anomalies were detected in this baby.

## Discussion

There is a frequent association between abdominal pregnancy and fetal malformations due to oligohydramnios. These include facial and cranial asymmetry, thoracic malformations, joint abnormalities, limb deformity, and central nervous system malformations. It is also associated with a peaked maternal mortality rate due to massive and grievous hemorrhage from the placental implantation site. The decision whether the placenta should be removed or left in situ should base on the size of the placental attachment to the intestine, the extent of omental involvement, and the surgeon's expertise [2].

Risk factors for developing abdominal pregnancy are a history of tubal pregnancy, dilatation and curettage, uterine surgeries, and artificial insemination. The common symptoms of an uncomplicated abdominal pregnancy are persistent abdominal pain, gastrointestinal symptoms like nausea and vomiting, painful fetal movements, general malaise, and altered bowel movements. Our patient suffered from frequent abdominal pain throughout the pregnancy. The typical physical signs due to its location in the peritoneal cavity are abdominal tenderness, an abnormal fetal lie, easily palpable fetal parts, and a displaced uterine cervix. This patient presented with an unstable lie. Diagnostic ultrasound criteria of abdominal pregnancy are a fetus in an extrauterine gestational sac, failure to see a uterine wall between the fetus and urinary bladder, a close approximation of the fetus to the maternal abdominal wall, localization of the placenta outside the confines of the uterine cavity, and an empty uterine cavity with no sign of ectopic tubal pregnancy [4].

Anatomical derangements usually complicate fetal delivery and enhance the intraoperative injury risk. A thorough postpartum inspection of placental implantation must be carried out to exclude the possibility of highly vascular adjacent parts, even though there is no apparent involvement of major vessels. Therefore, the objectives of surgical intervention are the delivery of the viable fetus and careful assessment and management of placental implantation without inducing hemorrhage [5].

In most reported cases, the placenta was found to be attached to adjacent organs or vessels. It was left intraabdominal to avoid hemorrhages or perforations caused by unwise attempts to separate the placenta [6]. In our case, the placenta was attached to the greater omentum and could be easily removed by omentectomy. Traditional practice was to leave the placenta in situ to undergo spontaneous resorption, and methotrexate was given to enhance the process. Leaving the placenta in situ will reduce the risk of hemorrhage, but it increases the risk of necrosis, pelvic abscess, and wound dehiscence [7].

In this case, the abdominal pregnancy was diagnosed on the operation table. There was no facility for ultrasonography in that district-level hospital and center for antenatal check-ups at that time. As the district was a remote area and the communication with the tertiary hospital was very poor, the patient was not sent for any ultrasonography. Similar instances can be seen in other underdeveloped countries [2]. Although the health amenities were poor, the outcome of this abdominal pregnancy was not similar to most cases where the baby died [2,3]; it was rather one of those rare cases where the baby was discharged healthy [8,9]. The mother of the baby was also healthy in contrast to other cases [9].

## Conclusions

Abdominal pregnancy is a very rare ectopic pregnancy that can be potentially serious due to its hemorrhagic complications. Therefore, along with the gynecologist, the availability of trained personnel, such as anesthetists, pediatricians, and general surgeons, is necessary for successful management. Even though our patient's pregnancy reached 38 weeks' gestation, no complications developed for her and her baby, and it was a miracle in a poor health setup like this case. Hence, the baby can rightly be termed a 'Miracle Baby'.

## References

[1] Mengistu Z, Getachew A, Adefris M (2015). Term abdominal pregnancy: a case report. J Med Case Rep.

[2] Nkusu Nunyalulendho D, Einterz EM (2008). Advanced abdominal pregnancy: case report and review of 163 cases reported since 1946. Rural Remote Health.

[3] Nassali MN, Benti TM, Bandani-Ntsabele M, Musinguzi E (2016). A case report of an asymptomatic late term abdominal pregnancy with a live birth at 41 weeks of gestation. BMC Res Notes.

[4] Dabiri T, Marroquin GA, Bendek B, Agamasu E, Mikhail M (2014). Advanced extrauterine pregnancy at 33 weeks with a healthy newborn. Biomed Res Int.

[5] Tolefac PN, Abanda MH, Minkande JZ, Priso EB (2017). The challenge in the diagnosis and management of an advanced abdominal pregnancy in a resource-low setting: a case report. J Med Case Rep.

[6] Godyn JJ, Hazra A, Gulli VM (2005). Subperitoneal placenta accreta succenturiate in the case of a successful near-term extrauterine abdominal pregnancy. Hum Pathol.

[7] Worley KC, Hnat MD, Cunningham FG (2008). Advanced extrauterine pregnancy: diagnostic and therapeutic challenges. Am J Obstet Gynecol.

[8] Gure T, Sultan S, Alishum R, Ali A, Dibaba B, Usmael I, Tsegaye S (2021). Term abdominal pregnancy with live baby: case report from Hiwot Fana Specialized University Hospital, Eastern Ethiopia. Int Med Case Rep J.

[9] Hailu FG, Yihunie GT, Essa AA, Tsega WK (2017). Advanced abdominal pregnancy, with live fetus and severe preeclampsia, case report. BMC Pregnancy Childbirth.

